# Holistic care in healthy aging: Caring for the wholly and holy human

**DOI:** 10.1111/acel.14021

**Published:** 2023-10-24

**Authors:** Lealani Mae Y. Acosta, E. Wesley Ely

**Affiliations:** ^1^ Critical Illness, Brain Dysfunction, and Survivorship (CIBS) Center Vanderbilt University Medical Center Nashville Tennessee USA; ^2^ Tennessee Valley Veteran's Affairs Geriatric Research Education Clinical Center (GRECC) Nashville Tennessee USA

**Keywords:** aging, goals of care, physician‐patient relationship, religion, spirituality

## Abstract

Health care should address the holistic gap between health outcomes, spirituality, religion, and humanistic care to optimize patient care. Treating the whole person encompasses both physical and metaphysical elements. Patients want health care professionals to recognize their spiritual and religious preferences, because these matter in their approach to illness, coping, and long‐term outcomes.

## Graphical abstract

Patient‐centered and humanistic care should respect patient preferences regarding spirituality and religion. Spiritual and religious practices can be positively correlated with mental health, social support, and health outcomes, especially among older people. Physicians and health professionals should be comfortable addressing spiritual questions through obtaining a spiritual intake and should recognize the value of the interdisciplinary team, including those specifically trained to address religious and spiritual matters.

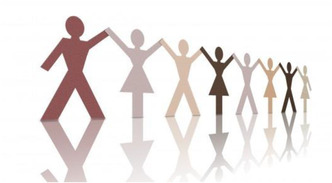

**Abbreviations**
FICA: Faith/Beliefs, Importance, Community Address in care or action
HOPE: Hope, Organized religion, Personal spirituality, Effects of care and decision
SNAP: Spiritual Needs Assessment for Patients
SPIRIT: Spiritual belief system, Personal Spirituality, Integration, Rituals/restrictions, Implications, and Terminal events

## 1 INTRODUCTION

“I would suggest that one of the fundamental reasons why so many doctors become cynical and disillusioned is precisely because, when the abstract idealism has worn thin, they are uncertain about the value of the actual lives of the patients they are treating. This is not because they are callous or personally inhuman: it is because they live in and accept a society which is incapable of knowing what a human life is worth.” *A Fortunate Man*, by John Berger (pp 165‐166) (Berger, 1997)

As physicians and providers of health care more generally, we are trained to identify and treat diseases that affect the human body. At the same time, we seek to “treat the patient, not the illness” and not reduce our patients merely to a set of organs and bodily functions (Ely, n.d.). We seek a unified diagnosis from various lab results and scans and a therapeutic plan that is tailored to the individual patient. Complementary to the eradication of pathology is the preservation of health and the prevention of disease.

In our focus on disease, we can lose sight of what it means to be healthy. If being ill makes us ill at ease, “dis‐eased,” then what entails being simply at “ease” or healthy? The etiology of the term “health” is related to other words like “whole” and “holy” (Berry, 1994). If we know that treating patients holistically means more than attending to their corporeal ailments, what else do we as physicians need to attend to so that we can treat the whole person?

In other words, what does it mean to provide patient‐centered care? The National Academy of Medicine defines “patient‐centered care” as being “respectful of, and responsive to, the individual patient preferences, needs, and values, and ensuring that patient values guide all clinical decisions” (Tinetti et al., 2019). Patients want their physicians to be cognizant of how they cope with disease, such as their goals and emotional responses, and desire a more holistic and humanistic approach, which should include the patients' spirituality and religion (Best et al., 2015; Fuchs et al., 2021; Puchalski et al., 2009).

We must begin by understanding these terms of “spirituality” and “religion.” A consistent definition of spirituality is challenging to identify across the literature. Spirituality has been defined as the “aspect of humanity that refers to the way individuals seek and express meaning and purpose and the way they experience their connectedness to the moment, to self, to others, to nature, and to the significant or sacred” (Puchalski et al., 2009). The definition of religion “[i]nvolves beliefs, practices, and rituals related to the transcendent, where the transcendent is God, Allah, HaShem, or a Higher Power in Western religious traditions, or to Brahman, manifestations of Brahman, Buddha, Dao, or ultimate truth/reality in Eastern traditions” (Koenig et al., 2012). Religion consists of beliefs and practices that each utilizes to address these spiritual approaches (Anandarajah & Hight, 2001).

For people whose experience of structured religion has been negative or unsatisfying, spirituality can be a welcome alternative; those without either spiritual or religious perspectives also need to be understood. For some, being “spiritual” is their way of seeking the deeper, numinous presence of a higher power without having to remain within what they perceive as a set of rules. Religious people may argue that for them, the formality of religion is liberating. Either way, it serves us well in this discussion to respect both as perfectly acceptable paths toward fulfillment and healing. While spirituality has lots in common with other similar concepts such as humanism, values, and morals, it connects more deeply to the sacred and the transcendent. “The transcendent is that which is outside of the self, and yet also within the self. […] Spirituality is intimately connected to the supernatural, the mystical, and to organized religion, although it also extends beyond organized religion” (Koenig et al., 2012). While distinct from a patient's values and beliefs, spirituality often undergirds these patient perspectives. There are patients who do not have either a specific religious or spiritual approach, and physicians must also acknowledge and respect the beliefs of those who are non‐affiliated or atheist.

Spirituality and religion are facets of patient‐centered care, which is of particular interest to older patients, who are more likely to utilize health care services with advancing years. In striving to construct “age‐friendly health systems,” we recognize that older patients can have a different set of perspectives and priorities from younger ones. For example, in contrast to younger patients, aging patients place greater emphasis on the “4Ms”: “What Matters, Mentation, Medication, and Mobility” (Cacchione, 2020; Institute of Healthcare Improvement, 2019; Lesser et al., 2022). Most relevant to the topic of holistic care is what “matters” to the aging adult patient, such as care preferences and outcome goals. Physicians need to identify what these patients deem significant, such as language, cultural, and religious preferences (Institute of Healthcare Improvement, 2019), to ensure personalized and age‐friendly care.

When considering the care of older patients, another “M” is relevant: mortality. Religious and spiritual practices are often positively correlated with factors such as mental health and decreased mortality. The Nurses' Health Study, an American study surveying nurses every 4 years for almost 20 years, found that women who attended religious services had a lower risk of cardiovascular and cancer mortality. While acknowledging the possible influence of covariates such as depression and smoking, the authors found that women who attended religious services more than once a week had 33% lower all‐cause mortality risk compared to those who never attended (Li et al., 2016). Another compelling example is from research on Black men in the United States, a historically burdened and marginalized group with shorter life expectancy compared to other demographic groups, which identified that more frequent participation in religious services had a 47% reduction in all‐cause mortality compared to peers without such involvement (Bruce et al., 2022).

Beyond longevity, improving quality of life and modifiable health outcomes is a priority for the aging population. Regardless of culture or geography, religiosity and spirituality are expressed in myriad ways across the globe and are increasingly reported by patients of advanced age. Spiritual engagement is positively associated with amelioration of common medical problems, such as myocardial infarctions, liver disease, mental health, and even shorter hospitalizations. The salubrious effects of social support, promotion of healthy behaviors, and decrease in stress are proposed mechanisms inherent in such practices (Zimmer et al., 2016). For example, research on patients with HIV/AIDS is a notable illustration of the positive benefits of religion/spirituality on health outcomes, such as pain and anxiety (Harding et al., 2005), higher social support, less depression, less use of alcohol and other recreational substances (Doolittle et al., 2021), better CD4 cell count, and longer survival (Doolittle et al., 2018).

The positive aspects of religion, spirituality, and health are not universally demonstrated. For example, the reverse causation may be more salient in that those patients who are in better health have an easier time attending worship services and may be more motivated to do so. Some research has demonstrated no association between religious practice and health (Speed, 2022). Some studies show a negative effect. For example, some patients may have negative coping ascribed to religious practices, such as spiritual or religious discontent or fearing divine punishment (Pargament et al., 1998). Such responses have been correlated with increased suicidal ideation in cancer patients (Trevino et al., 2014). Religious and moral distress can also arise from those struggling with competing or seemingly incompatible belief systems, which are correlated with worsened depression and anxiety (Abu‐Raiya et al., 2015). Clinicians should be attuned to the potentially harmful effects of patients' religious and spiritual perspectives.

Religiosity and spirituality are matters that are deeply relevant to in a humanistic perspective in the medical care (Ely, n.d.; Best et al., 2015; Koenig et al., 2017; Puchalski et al., 2009). What does it mean to have a humanistic approach to medicine? Humanism in medicine, defined as “infusing and sustaining our healthcare system with a culture of compassion, caring, and respect for patients and practitioners,” overlaps with spirituality and religion as far as the goal of “provision of compassionate care” (Puchalski et al., 2014). This concept of humanism fosters “a compassionate, caring, familiar, and empathic relationship,” which can promote positive behaviors such as “respect, caring, integrity, and service.” Medical training necessarily emphasizes the science of clinical care. It is the art of medicine and the fostering of that patient–physician relationship by espousing such values that enable such care to transpire (Gold & Gold, 2006).

If patients seek medical help for physical healing, do they want doctors to address these more metaphysical matters? For many patients, the answer is yes (Best et al., 2015; Puchalski et al., 2009; Zimmer et al., 2016). At least half of patients feel it is appropriate for a doctor to ask about spiritual needs in at least some circumstances. Patients were asked across a spectrum of health care situations, both inpatient and outpatient, chronic and life‐threatening, as well as religious affiliations, including those who denied having religious beliefs (Best et al., 2015). While research has historically focused less on spirituality and more on religiosity because it is easier to quantify, attitudes about the meaning of life and transcendent contemplation are common, even without specific religious practice (Zimmer et al., 2016). While not expecting spiritual advice from their physicians per se, patients want their medical team to understand about how they find meaning and cope with disease (Best et al., 2015). Certainly other key members of the interdisciplinary medical team, such as a chaplains (Best et al., 2015; Puchalski et al., 2009), who have been more specifically trained to address spiritual needs, can be involved. As physicians typically lead such medical teams, we need to be able to address spirituality, as emphasized by organizations like the American Association of Medical Colleges (Behan et al., 2022) and the World Health Organization (*Social Science & Medicine*, 1995). Research underscores the need to address these religious and spiritual matters as being core to the older patients' perspectives regarding healthcare (Institute of Healthcare Improvement, 2019), as older patients are generally more religious than the younger generation, though this does not hold absolutely across all cultures (Pew Research Center, 2018).

While patients want their doctors to know about their spiritual or religious beliefs, particularly when facing a serious illness (Balboni et al., 2010), physicians often do not inquire. A recent survey of U.S. physicians found that about 10% of them often inquired about spirituality and religion (Koenig et al., 2017). Such conversations often seem to arise during major life events, such as birth, major surgery (Best et al., 2015), serious illness (Balboni et al., 2010), and end‐of‐life care (Koenig et al., 2017). Yet these matters affect patients of every age and type of illness: acute or chronic, severe or minor, routine or complex. We need to understand how these perspectives affect myriad aspects of patient care, such as medical decision‐making and finding meaning in their health struggles (Best et al., 2015), especially as they age (Lesser et al., 2022).

In our practice of medicine, we are urged to recognize and respect what matters to patients. While outlined in a model constructed for the purposes of addressing age‐friendly health systems (Lesser et al., 2022), such interests span across all ages when we consider communicating with patients and consideration of outcomes and planning, though this may become a higher priority as patients grow older. The biopsychosocial–spiritual model of care encapsulates this approach, which incorporates “the biological, the psychological, the social, and the spiritual” as being intertwined and essential (Sulmasy, 2002).

## 2 OBTAINING A SPIRITUAL INTAKE

How does the humanistic physician begin to understand patients' spiritual beliefs, values, or needs? One step is to obtain a spiritual intake, as routinely as one might obtain a family or social history (Ely, n.d.). A “screening” spiritual intake can be as simple as taking a few minutes to ask “about the patient's religious or spiritual background if any, whether faith gives them hope, whether they have any spiritual beliefs that might influence medical decisions, or if other spiritual concerns are present that need to be addressed for health reasons” (Koenig et al., 2017). Beyond commonly recognized religious beliefs, such as Jehovah's Witness patients' beliefs that preclude administration of blood products (Chae et al., 2020), patients may have a spiritual outlook that can influence other major medical decisions, such as reproductive health, religion‐based alternative medical therapies (e.g., faith healing) (Maugans, 1996), and end‐of‐life care (Koenig et al., 2017). Perhaps more universally important, the patient's spiritual beliefs may affect their pattern of healing and what they need from the medical team in terms of existential and non‐medical support to accelerate healing. From a physician's perspective, it should not be underestimated how helpful this sort of whole‐person care can be of service to one's vocation and even to the prevention of burnout.

## 3 TOOLS FOR A SPIRITUAL INTAKE

Given the importance of a spiritual intake, the physician would benefit from structured tools that may be utilized to obtain such a history. Some of these include the HOPE (Hope, Organized Religion, Personal Spirituality, Effects of Care and Decision) (Anandarajah & Hight, 2001), FICA (Faith/Beliefs, Importance, Community Address in Care or Action) (Puchalski & Romer, 2000), SNAP (Spiritual Needs Assessment for Patients) (Sharma et al., 2012), and SPIRIT (Spiritual Belief System, Personal Spirituality, Integration, Rituals/Restrictions, Implications, and Terminal Events) assessments. These include questions about personal beliefs, such as, “Do you belong to any spiritual or religious group or community?” (Puchalski & Romer, 2000) or “What aspects of your spirituality or spiritual practices do you find most helpful to you personally?” (Anandarajah & Hight, 2001) Other queries focus more directly on patient care, such as, “What aspects of your religion/spirituality would you like me to keep in mind as I care for you?” (Maugans, 1996) Or “Are there any specific practices or restrictions I should know about in providing your medical care? (e.g., dietary restrictions, use of blood products) (Anandarajah & Hight, 2001).

## 4 INCORPORATING THE INTERDISCIPLINARY TEAM TO PROVIDE SPIRITUALLY INFORMED HEALTHCARE

Beyond history‐taking to determine the spiritual values of our patients, there are practical ways to help patients receive spiritual help and attain spiritual wellness that are within the purview of every clinician. If the patient says that they do have spiritual values, we can then follow up with them to address what those values are in a direct manner. Most commonly, this would be by asking the patient if he/she wants to speak with a hospital chaplain or if we can contact a spiritual advisor of his/her choosing (Best et al., 2015; Puchalski et al., 2009). This allows us to involve her/his specific religious community. Or, if the patient says that she/he is an atheist or agnostic, it allows us to show them respect to affirm that we will not speak to them about God but rather about whatever aspects of her/his life goals are preferred. Sometimes atheist and agnostic patients, we might add, do want to speak to a chaplain to discuss specific existential wishes and concerns (Behan et al., 2022). Taking this approach shows respect and love for the patient and demonstrates directly that we value the whole person.

It is not enough to care only for the physical aspects of our patients; directing our attention to getting specially trained spiritual advisors to the bedside shows how we value mental and spiritual dimensions of our patients as well. Most clinicians are not formally trained as religious professionals; thus, it is best to let patients lead the conversations regarding their specific religious needs. We can take part as far as we feel comfortable, but it is important to emphasize that we are not to proselytize at the bedside. This would be an abuse of the power differential of the white coat. It would, however, be appropriate for us to use this time with our patients as an opportunity for collaborative goal‐setting. We can ask her/him what matters in terms of overall goals of care and goals of their upcoming weeks and months. This is a direct way of speaking with them about specific opportunities to lift them up in their illness toward these goals.

An underlying theme of how best to address and respect patients' spirituality and religion is the trust necessary in the patient–physician relationship. Patients will not address these types of discussions unless they respect the physician, and both people in this relationship will need to feel empowered to broach spiritual topics before this conversation will likely take place (Best et al., 2015; Koenig et al., 2017). Yet it has to be initiated by one of the two, and data support that the majority of patients want their physicians to ask about their spiritual beliefs, even if it is to say, “Yes, I am an atheist, and please don't talk to me about God.” Indeed, some patients may not want to discuss spirituality, while others may prioritize it (Behan et al., 2022). It is very uncommon for a patient to be angered or in any way disgruntled by a neutral inquiry about whether or not they have spiritual values that are relevant to their healing process.

Beyond allowing patients to feel heard and recognized as individuals, respecting and recognizing patients' spirituality has also been associated with better outcomes, such as improved quality of life (Balboni et al., 2010), as patients' beliefs are associated with decreased mortality (Bruce et al., 2022; Li et al., 2016). Conversely, not addressing spirituality and religion has been correlated with patient perception of poorer quality of life (Balboni et al., 2010; Vallurupalli et al., 2012), poorer quality of care, and lower satisfaction with care (Astrow et al., 2007).

Ultimately, patients want to be treated as whole individuals beyond a pathologic entity (Ely, n.d.; Doolittle et al., 2018; Lesser et al., 2022), which becomes a higher priority to older patients (Lesser et al., 2022), including spiritual and religious beliefs that become increasingly important with age (Zimmer et al., 2016). Having a genuine, personal relationship with patients (Best et al., 2015; Koenig et al., 2017) helps foster that deeper understanding of what matters to them regarding their health, decision‐making, and lives (Lesser et al., 2022), including how perspectives may shift with age and approaching end‐of‐life care, both with healthy aging and in specific palliative or hospice health settings (Balboni et al., 2010; Harding et al., 2005; Koenig et al., 2017; Vallurupalli et al., 2012). As Wendell Berry, the writer and poet (Hart et al., 2003), succinctly stated, “In healing, the body is restored to itself” (Berry, 1994). The identity of being hale, or whole, encompasses both the physical and metaphysical within patient care, both for the receiver as the patient and for the physicians and other providers of health care entrusted to serve the patient.

**Summary table of recommendations**

**Summary**

1. Patient‐centered and humanistic care should respect patient preferences regarding spirituality and religion
2. Spiritual and religious practices can be positively correlated with mental health, social support, and health outcomes
3. Physicians and health professionals should be comfortable addressing spiritual questions with all patients, particularly older patients, such as by obtaining a spiritual intake
4. Physicians and healthcare professionals should recognize the value of the interdisciplinary team, including those specifically trained to address religious and spiritual matter, such as chaplains.

## AUTHOR CONTRIBUTIONS

Dr. Acosta and Dr. Ely both have made substantial contributions to conception and design, or acquisition of data, or analysis and interpretation of data; been involved in drafting the manuscript or revising it critically for important intellectual content; and given final approval of the version to be published. Each author should have participated sufficiently in the work to take public responsibility for appropriate portions of the content and agreed to be accountable for all aspects of the work in ensuring that questions related to the accuracy or integrity of any part of the work are appropriately investigated and resolved.

## FUNDING INFORMATION

None.

## CONFLICT OF INTEREST STATEMENT

No conflict of interest.

## Data Availability

Data sharing is not applicable to this article as no new data were created or analyzed in this study.
